# Biochemical and structural analyses of metallo-β-lactamase VIM-28: impact of substitutions at residues 224 and 228 on substrate profile, stability, and zinc affinity

**DOI:** 10.1128/spectrum.02788-25

**Published:** 2026-02-09

**Authors:** Hiromu Sato, Nao Ishizawa, Ryo-ya Koto, Kaisei Hiura, Hiyori Saito, Yoshiki Kato, Nancy D. Hanson, Yoshikazu Ishii, Akiko Shimizu-Ibuka

**Affiliations:** 1 Department of Applied Life Sciences, Niigata University of Pharmacy and Applied Life Sciences 222725 , Niigata, Japan; 2 Graduate School of Science, Kanagawa University 12853, Yokohama, Kanagawa, Japan; 3 Department of Medical Microbiology and Immunology, Creighton University 6216, Omaha, Nebraska, USA; 4 Department of Microbial Genomics and Ecology, Center for the Planetary Health and Innovation Science, The IDEC Institute, Hiroshima University 12801, Higashi-Hiroshima City, Hiroshima, Japan; Universidad de Buenos Aires, Buenos Aires, Argentina

**Keywords:** zinc, metallo-β-lactamases, antibiotic resistance

## Abstract

Metallo-β-lactamases (MBLs) hydrolyze a broad range of β-lactams, including carbapenems. VIM-28, an MBL identified in *Pseudomonas aeruginosa*, is an H224L/S228R variant of VIM-1 and H224L variant of VIM-4. Compared with VIM-26 (R228S), VIM-28 displayed decreased *K*_m_ (12.5 for VIM-28 vs 513 μM for VIM-26; 9.66 vs 150 μM) and increased *k*_cat_/*K*_m_(15.3 vs 1.81 μM^−1^s^−1^; 28.6 vs 5.89 μM^−1^s^−1^) for ampicillin and cephalothin, respectively. VIM-1, which has a His in position 224 and Ser in position 228, displayed intermediate kinetic values (*K*_m_ 215 and 77.0 μM; *k*_cat_/*K*_m_ 2.63 and 8.61 μM^−1^s^−1^) for ampicillin and cephalothin, respectively, indicating that the presence of a positively charged residue at either position 224 or 228 enhanced substrate interactions. The combined L224H/R228S substitutions in VIM-1 increased the catalytic efficiency of the enzyme for ceftazidime by more than one order of magnitude. These kinetic trends were consistent with the minimum inhibitory concentration (MIC) data, with an eightfold increase in ceftazidime MIC for VIM-1-producing cells. Moreover, relative MIC assay showed that VIM-26 (R228S)-producing cells were more refractory to the addition of chelators than cells producing VIM-28, whereas VIM-4 (L224H)-producing cells showed reduced resistance, suggesting that the residues at positions 224 and 228 influence the metal-binding affinity of the enzyme. Differential scanning fluorimetry assay revealed that the R228S substitution increased the melting temperature of the enzyme, whereas the L224H substitution reduced its thermal stability. VIM-28 exhibited high catalytic efficiency for substrates other than ceftazidime, and the H224L substitution conferred higher zinc-binding affinity and thermal stability compared with VIM-4.

**IMPORTANCE:**

β-Lactam-resistant bacteria, especially carbapenem-resistant strains, pose a major global health threat, often through metallo-β-lactamases (MBLs). To anticipate resistance evolution, we characterized VIM-28, a variant of the widespread VIM-1/VIM-4-type enzymes, focusing on the roles of two variable L10 loop residues. Substitutions at positions 224 and 228 strongly affected substrate specificity, enzyme stability, and zinc affinity. Arg228 was important for carbapenem recognition, while combined substitutions at positions 224 and 228 could enhance activity toward ceftazidime. Notably, the R228S substitution improved zinc binding and thermal stability, supporting enzyme function under zinc-limited host conditions. These findings reveal mechanisms driving MBL diversity and highlight evolutionary strategies sustaining antibiotic resistance.

## INTRODUCTION

β-Lactam antibiotics inhibit bacterial transpeptidases, key enzymes involved in bacterial peptidoglycan biosynthesis, and have been widely used to treat bacterial infections. However, the rise in antibiotic use has caused an increase in β-lactam resistance, posing a serious threat to global public health. In gram-negative bacteria, the most common mechanism of resistance against β-lactam antibiotics is the production of β-lactamases. These enzymes inactivate β-lactams by hydrolyzing the amide bond in the four-membered azetidinone ring (1, 2).

β-Lactamases are categorized into two major classes based on their catalytic mechanisms: serine β-lactamases (SBLs) and metallo-β-lactamases (MBL) (2–4). MBLs feature a conserved αβ/βα sandwich-fold structure, with their active site located in the central groove on the edge of the two β-sheets. One or two zinc ions bound to the active site are essential for catalysis (3). MBL production in resistant bacteria poses a substantial threat to antibiotic therapy because of their broad substrate specificity, hydrolyzing all classes of β-lactams except monobactams. Furthermore, treatment options remain limited, although boronate-based inhibitors have been developed and are currently undergoing clinical trials (4–8).

MBLs are further divided into three subclasses, namely, B1, B2, and B3, based on their primary zinc coordination shell and amino acid sequence. Subclass B1 enzymes possess a binuclear zinc center and include many clinically relevant MBLs, such as New Delhi MBLs (NDMs), imipenemases (IMPs), and Verona integron-encoded MBLs (VIMs). VIM and IMP enzymes are disseminated worldwide through within integron structures (3, 4, 9). As of August 2025, more than 90 variants of VIM enzymes have been identified (https://www.ncbi.nlm.nih.gov/pathogens/refgene/, http://www.bldb.eu/) (10). These variants are classified into up to five clusters, with the major clusters including VIM-2 and VIM-1/VIM-4. VIM-2 is the most prevalent VIM-type enzyme and is primarily associated with *Pseudomonas aeruginosa*, whereas VIM-1/VIM-4-like enzymes are more frequently reported in *Enterobacterales* (1, 9, 11). The VIM-1 and VIM-2 amino acid sequences diverge by 7%. VIM-2 hydrolyzes carbapenems more efficiently but is more susceptible to inactivation by chelators than VIM-1 (12).

Position 224 is occupied by Lys in many B1 MBLs, and it is critical for binding the C3/C4 carboxyl group of the substrate (11). The residues at positions 224 and 228 in the L10 loop vary among VIM enzymes. Lys224 is not conserved in VIM enzymes and is often replaced with His in VIM-1/VIM-4–like enzymes and with Tyr in VIM-2-like enzymes. When a Lys is not present at position 224, the Arg at position 228 defines a positive charge in that space for substrate binding in VIM-2. This Arg residue is not conserved in all VIM enzymes, and position 228 is occupied with Ser in VIM-1 (13) (Table 1).

**TABLE 1:** Amino acid conservation in VIM enzymes

Cluster	Enzyme	Residue										
		148	215	223	224	228	248	251	257	258	301	304
VIM-1/VIM-4	VIM-1	Ala	Asn	Val	His	Ser	Val	Lys	Glu	Val	Gln	Ala
	VIM-4	Ala	Asn	Val	His	Arg	Val	Lys	Glu	Val	Gln	Ala
	VIM-28	Ala	Asn	Val	Leu	Arg	Val	Lys	Glu	Val	Gln	Ala
	VIM-26	Ala	Asn	Val	Leu	Ser	Val	Lys	Glu	Val	Gln	Ala
VIM-2	VIM-2	Val	Ser	Ile	Tyr	Arg	Ile	Gln	Gln	Phe	Lys	Thr
	VIM-20	Val	Ser	Ile	Tyr	Arg	Ile	Gln	Gln	Phe	Lys	Thr
VIM-7	VIM-7	Ala	Arg	Val	His	Arg	Ile	Gln	Glu	Val	Gln	Thr

VIM-28 is a VIM-1/VIM-4-type enzyme carrying H224L and S228R substitutions relative to VIM-1 and an H224L substitution relative to VIM-4. The gene encoding VIM-28 was identified in a *P. aeruginosa* isolate from Egypt (GenBank ID: JF900599) (14). In this study, we analyzed the kinetic properties and thermal stability of VIM-28, together with related VIM-type enzymes (VIM-1, VIM-4, and variants carrying substitutions at positions 224 and/or 228), as well as the crystal structure of VIM-28 and the susceptibility profiles of *E. coli* cells producing these enzymes. Overall, our results demonstrate that VIM-28 not only exhibits high catalytic activity toward a broad range of antibiotics but also shows higher zinc-binding affinity and thermal stability than VIM-4, thereby conferring enhanced resistance to zinc deprivation in producing cells.

## RESULTS

In this study, we compared kinetic properties and thermal stability of VIM-28 (Leu224 and Arg228) with those of VIM-1 (His224 and Ser228), VIM-4 (His224), and VIM-26 (Ser228). In addition, we analyzed VIM-28 variants carrying V223I/L224Y substitutions, considering that VIM-2-type enzymes have Ile residue at position 223 and a Tyr residue at position 224. We also examined the susceptibility profiles of *E. coli* cells producing these enzymes.

### Kinetic properties of the VIM-28

The results of the steady-state kinetic analysis are shown in Table 2. The *k*_cat_ values were not corrected for the fraction of Zn-bound enzyme, as the Zn content of each enzyme preparation was not measured in this study. Few differences were observed in kinetic parameters between VIM-28 and VIM-4, although VIM-4 showed moderately higher catalytic efficiency (*k*_cat_/*K*_m_) for cephalothins and meropenem. V223I/L224Y substitution in VIM-28 caused minor changes in the kinetic properties, reducing both *k*_cat_ and *K*_m_ values for penicillin G, cefotaxime, and meropenem, by up to twofold, and increasing *k*_cat_/*K*_m_ toward ceftazidime by approximately twofold. The V223I/L224Y/V248I triple variant decreased the *k*_cat_ values for penicillins, cefotaxime, and meropenem by approximately 1.7-fold, whereas it increased the *k*_cat_/*K*_m_ value by 2.7-fold toward ceftazidime. The increase of *k*_cat_ and *K*_m_ values for cephalothin and cefotaxime observed in VIM-4 was not observed in these variants.

**TABLE 2:** Kinetic parameters of VIM-28 and its related enzymes

	VIM-1 (L224H/R228S)			VIM-4 (L224H)			VIM-26 (R228S)		
	*k*_cat_ (s^−1^)	*K*_m_ (μM)	*k*_cat_/*K*_m_ (s^−1^μM^−1^)	*k*_cat_ (s^−1^)	*K*_m_ (μM)	*k*_cat_/*K*_m_ (s^−1^μM^−1^)	*k*_cat_ (s^−1^)	*K*_m_ (μM)	*k*_cat_/*K*_m_ (s^−1^μM^−1^)
Ampicillin	565 ± 54	215 ± 23	2.63	197 ± 19	25.7 ± 2.2	7.67	931 ± 124	513 ± 28	1.81
Penicillin G	752 ± 35	329 ± 33	2.29	812 ± 34	93.9 ± 9.3	8.65	NA^*a*^	>500	0.630
Cephalothin	663 ± 39	77.0 ± 5.5	8.61	756 ± 64	18.8 ± 1.0	39.9	884 ± 37	150 ± 7	5.89
Cefotaxime	588 ± 29	134 ± 9	4.39	651 ± 43	41.8 ± 2.7	15.6	NA	>400	2.25
Ceftazidime	NA^*a*^	>400	0.168^*b*^ ± 0.012	NA^*a*^	>400	0.0441^*b*^ ± 0.0030	NA^*a*^	>400	0.0339^*b*^ ± 0.0035
Meropenem	75.5 ± 1.9	339 ± 36	0.222	17.0 ± 1.3	8.35 ± 0.39	2.04	90.0 ± 4.8	241 ± 27	0.393

	VIM-28			V223I/L224Y			V223I/L224Y/V248I		
	*k*_cat_ (s^−1^)	*K*_m_ (μM)	*k*_cat_/*K*_m_ (s^−1^μM^−1^)	*k*_cat_ (s^−1^)	*K*_m_ (μM)	*k*_cat_/*K*_m_ (s^−1^μM^−1^)	*k*_cat_ (s^−1^)	*K*_m_ (μM)	*k*_cat_/*K*_m_ (s^−1^μM^−1^)
Ampicillin	191 ± 36	12.5 ± 4.9	15.3	191 ± 19	19.5 ± 3.6	9.80	201 ± 45	22.4 ± 7.5	9.00
Penicillin G	1240 ± 80	176 ± 12	7.03	567 ± 16	121 ± 10	4.69	482 ± 21	118 ± 25.	4.09
Cephalothin	276 ± 13	9.66 ± 0.79	28.6	258 ± 7	7.73 ± 0.54	33.4	248 ± 16	7.80 ± 0.73	31.8
Cefotaxime	285 ± 9	27.2 ± 1.8	10.5	169 ± 6	24.0 ± 3.8	7.05	155 ± 7	24.8 ± 5.0	6.25
Ceftazidime	NA^*a*^	>400	0.0359^*b*^ ± 0.0034	NA^*a*^	>400	0.0834^*b*^ ± 0.0169	NA^*a*^	>400	0.0966^*b*^ ± 0.015
Meropenem	20.6 ± 1.2	14.3 ± 0.9	1.44	9.12 ± 0.26	7.21 ± 0.88	1.26	7.71 ± 0.50	8.17 ± 0.88	0.944

^*a*^ Not available; *K*_m_ too high to determine accurately.

^*b*^ The *k*_cat_/*K*_m_ ratio was calculated from the initial slope using *v* = *k*_cat_/*K*_m_([S]).

Based on the high *K*_m_ values, the catalytic efficiency of the enzymes with R228S substitution was lower than that of VIM-28 for most of the substrates tested in this study. The increase of *K*_m_ values for penicillins, cephalothin, and cefotaxime in VIM-26 was greater than that observed in VIM-1, which severely decreased the catalytic efficiency in VIM-26. VIM-26 and VIM-1 showed decreased catalytic efficiency toward meropenem by fourfold and sixfold, respectively. It was again due to increased *K*_m_ values, indicating that the Arg residue at position 228 is critical for meropenem recognition and/or binding. In contrast, the VIM-1 exhibited fivefold higher catalytic efficiency than VIM-28 for ceftazidime. However, increased *k*_cat_/*K*_m_ was not observed in VIM-26, indicating that the combined R228S and L224H substitutions were more suitable for efficient ceftazidime hydrolysis.

### Susceptibility profiles

MICs were determined using *E. coli* DH5α cells harboring the plasmids containing VIM-encoding genes in pMW119_Kan^R^_Ptac, a low-copy cloning vector constructed from pMW119 (Table 3). While little difference was observed in the MICs for the penicillins, cephalothin, and cefotaxime, the cells producing VIM-1 (L224H/R228S) showed an eightfold increase in ceftazidime MICs compared with the VIM-28, and a 16-fold increase compared with VIM-4 (L224H). Furthermore, the cells producing VIM-28 had a fourfold increase in meropenem MICs compared with the cells producing any other enzymes tested.

**TABLE 3:** Antibiotic susceptibility of *Escherichia coli* DH5α harboring the expression plasmid for VIM-28 and its related enzymes

	MIC (µg/mL)						
Variant^*a*^	Ampicillin	Penicillin G	Cephalothin	Cefotaxime	Ceftazidime	Meropenem	Aztreonam
DH5α^*b*^	2	16	8	<0.125	0.125	<0.03125	0.03125
Control^*c*^	2	16	16	<0.125	0.125	<0.03125	0.125
VIM-28	>2,048	>2,048	1,024	128	64	8	0.0625
VIM-4 (L224H)	2,048	>2,048	256	64	32	2	0.125
VIM-26 (R228S)	2,048	2,048	512	64	128	2	0.03125
VIM-1 (L224H/R228S)	2,048	>2,048	1,024	128	512	2	0.125
V223H/L224Y	2,048	2,048	1,024	128	128	2	0.25

^*a*^ Variants were all expressed in *E. coli* DH5α.

^*b*^ *E. coli* DH5α with no plasmid.

^*c*^ *E. coli* DH5α with an expression vector with no b-lactamase gene insertion (pMW119_Kan^R^_Ptac).

**Fig 1:**
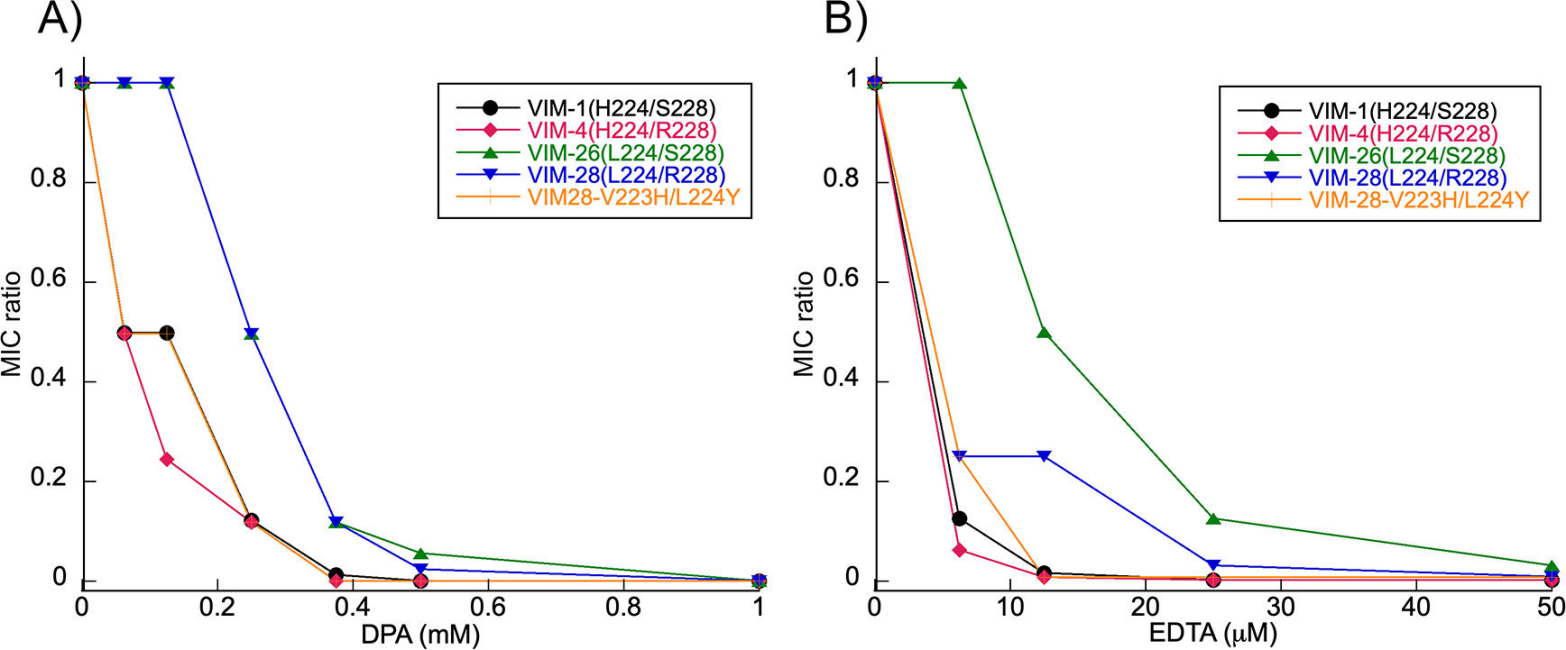
Relative MIC values of cefotaxime for *E. coli* producing VIM-28 and related VIM enzymes in medium supplemented with different concentrations of (**A**) DPA and (**B**) EDTA.

To evaluate the resistance conferred by VIM-28 and related enzymes to cefotaxime in the presence of the metal-chelating agents DPA and EDTA, relative MICs were calculated according to a previously described method (15) and compared among cells producing these enzymes. Overall, the trends in relative minimum inhibitory concentration (MIC) changes caused by substitutions at positions 223, 224, and 228 were similar in the presence of either DPA or EDTA. However, EDTA exerted comparable effects at concentrations more than one order of magnitude lower than DPA. In the presence of DPA, VIM-26-producing cells showed resistance comparable to VIM-28-producing cells, whereas they were more refractory to EDTA than the VIM-28-producing cells. VIM-4-, VIM-1-, and V223H/L224Y-producing cells showed reduced resistance in the presence of either chelator (Fig. 1).

### Thermal stability of VIM-28 and its related enzymes

The melting temperatures (*T*_m_) of VIM-28 and its related enzymes were determined by differential scanning fluorimetry (DSF) using zinc concentrations of 0–50 μM (Table 4; Fig. S1). *T*_m_ was highest in the absence of zinc ions across all enzymes and decreased with increasing zinc concentration. In the absence of additional zinc ions, VIM-26 had the highest *T*_m_ (65.5°C), followed by VIM-28 (62.0°C). VIM-4 and VIM-1 had a lower *T*_m_, with VIM-4 showing the lowest *T*_m_ (54.5°C). The *T*_m_ of the V223I + L224Y variant was also lower than VIM-28. These results indicated that the R228S substitution increases the thermal stability of the enzyme, whereas the Leu224 substitution reduced it.

**TABLE 4:** Melting temperature (*T*_m_) of VIM-28 and its related enzymes analyzed by differential scanning fluorimetry assays under different zinc concentrations^*a*^

		Enzymes				
	Zinc concentration (mM)	VIM-28	L224H (VIM-4)	R228S (VIM-26)	V223I/L224Y	L224H/R228S (VIM-1)
*T*_*m*_ (°C)	0	62	54.5	65.5	59.5	58.0 (50.0)
	10	61	53	64.5	59	58.0 (47.5)
	50	60.0 (54)	48.5	63.5	55.5	54.5

^*a*^ When two stages of denaturation are observed, the lower denaturation temperature is indicated in parentheses.

### Three-dimensional structural analysis of VIM-28

**Fig 2:**
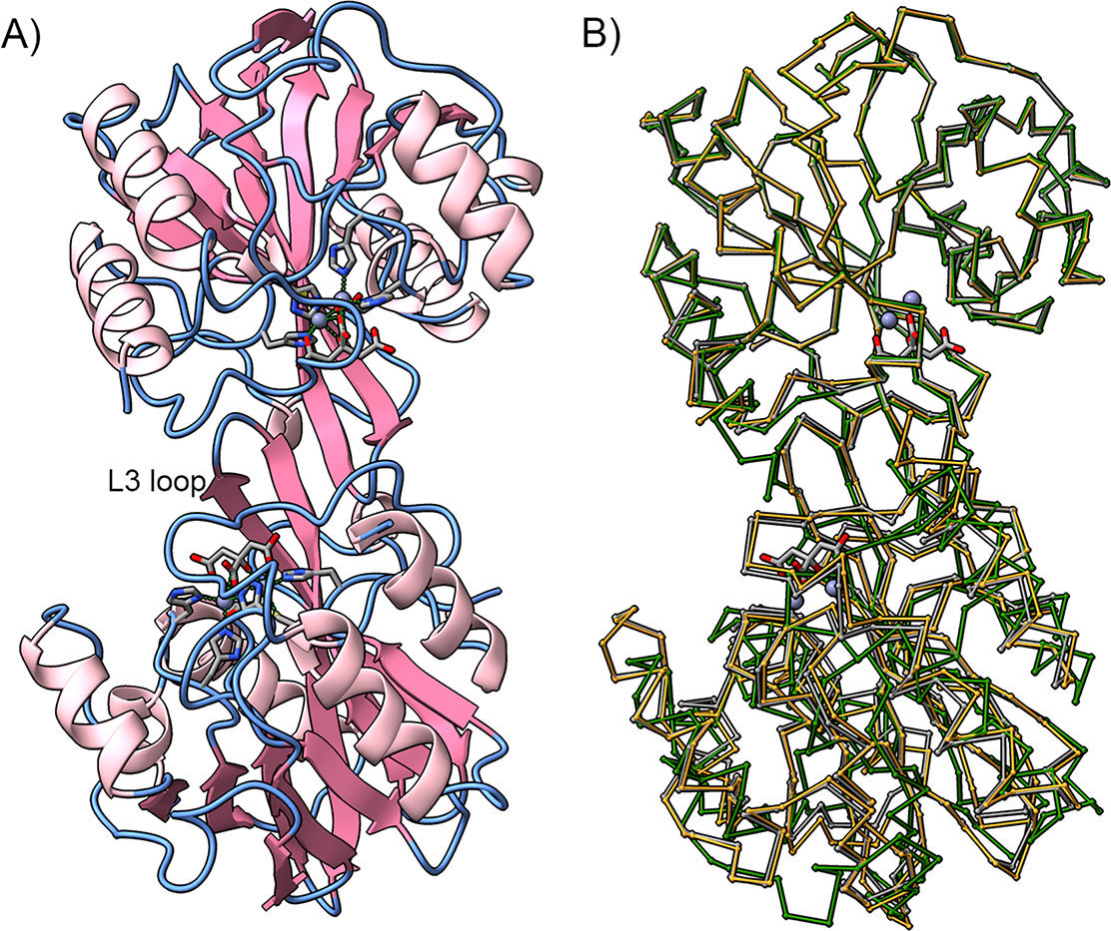
Intermolecular interaction in the crystal structure of VIM-28. In all the panels, the zinc ions are shown as gray spheres, while their ligand residues/molecules are shown as stick models. (**A**) Two VIM-28 molecules form a pseudo-dimer in an asymmetric unit. Two β-hairpin structures with an L3 loop from each molecule form a four-stranded antiparallel β-sheet at the interface. (**B**) Ca traces of VIM-28 (gray), VIM-2 (5LSC, green), and VIM-4 (2WHG, orange) in the same orientation as VIM-28 in panel **A**. The upper molecules are superimposed. The figure was created using ChimeraX (16).

The crystal structure of VIM-28 was determined at 2.0 Å (PDB code 7YRP, Table S1). It contains two molecules (A and B) in an asymmetric unit. Molecule A includes residues 31–259, while molecule B includes residues 29–259. The two molecules were in a pseudo-twofold symmetrical relationship. The β-hairpin structure containing the L3 loop (residues 60–66) of each molecule aligns to form a four-stranded antiparallel β-sheet in the crystal packing (Fig. 2A). The same β-sheet formation was observed in the crystal structures of VIM-4 (PDB: 2WHG), VIM-2 (PDB: 5LSC), VIM-5 (PDB: 5A87), and oxidized VIM-31 (PDB: 4FSB) (Fig. 2B). The root mean square deviation (RMSD) for Cα between the molecules A and B is 0.11 Å. The superposition of the VIM-28 model (molecule A) with those of the other VIM enzymes produced the following RMSD values: 0.20 Å with VIM-4 (molecule A) for 1,415 atoms; 0.294 Å with VIM-1 (PDB entry: 5N5G) for 1,370 atoms; and 0.23 Å with VIM-2 (PDB entry: 4BZ3) for 1,219 atoms. Only minor differences were observed in the overall structure between VIM-28 and the other VIM structures.

**Fig 3:**
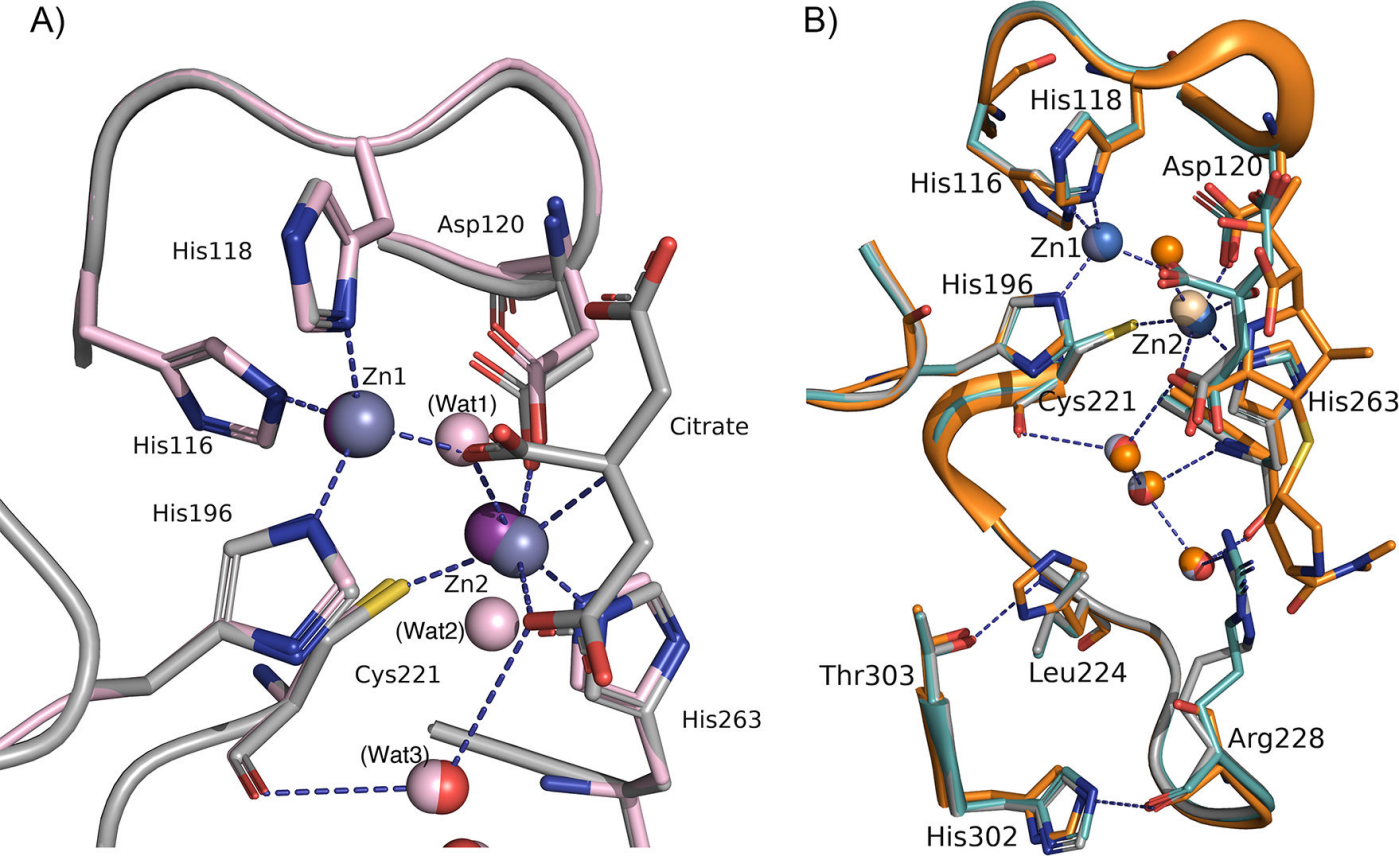
Active site of VIM-28. (**A**) The main chain and carbon atoms of molecule A are shown in gray. The active site of VIM-28 (molecule A) is compared with that of VIM-1 (PDB: 5N5G). The main chain, carbon atoms, and water molecules of VIM-28 are shown in gray, whereas those of VIM-1 are shown in pink. The zinc ions bound to the VIM-1 active site are shown in purple. (**B**) Substrate-binding cavity of VIM-28. Molecules A and B are shown in gray and cyan, respectively. The zinc ions and the water molecules of molecule A are shown in gray, blue, and red, respectively. Hydrogen bonds in molecule A are shown as dotted lines. The structure of VIM-1 with hydrolyzed meropenem (shown in orange, PDB: 5N5I) was superimposed on the structure of VIM-28. The figure was created using PyMOL (Schrödinger, LLC, NY, USA).

The active site of VIM-28 contains one citrate anion and two zinc ions, Zn1 and Zn2 (Fig. 3A). The distance between Zn1 and Zn2 is 3.9 Å in both molecules A and B. The geometry of the Zn1 site is tetrahedral, while the Zn2 ion is octahedral, which was also observed in other VIM enzymes, such as VIM-1 and VIM-2 (17, 18). A carboxylate oxygen of the citrate anion replaces the water molecule that bridges the two zinc ions in VIM enzymes (Wat1 in VIM-1 structure, Fig. 3A). The octahedral coordination of Zn2 showed that two citrate oxygen atoms instead of water molecules coordinate Zn2 (Wat1 and Wat2) in the VIM-1 structure. Cys221 coordinated with Zn2 and was refined as a reduced form in VIM-28. The electron density of Zn2 is weaker than that of Zn1, and the occupancy of Zn2 is 0.5 on structural refinement.

The Arg228 side chain protrudes into the substrate-binding cavity as part of the hydrogen bonding network formed in the active site via the water molecules (Fig. 3B). The main chain NH of Leu224 is hydrogen-bonded to the side chain of Thr303. Structural comparison of VIM-28 and VIM-1 complexed with hydrolyzed meropenem indicates that the side chain of Arg228 is positioned very close to the R2 group of the substrate (Fig. 3B).

### Effect of amino acid substitutions between VIM-1 and VIM-2 clusters on the property of the enzyme

**Fig 4:**
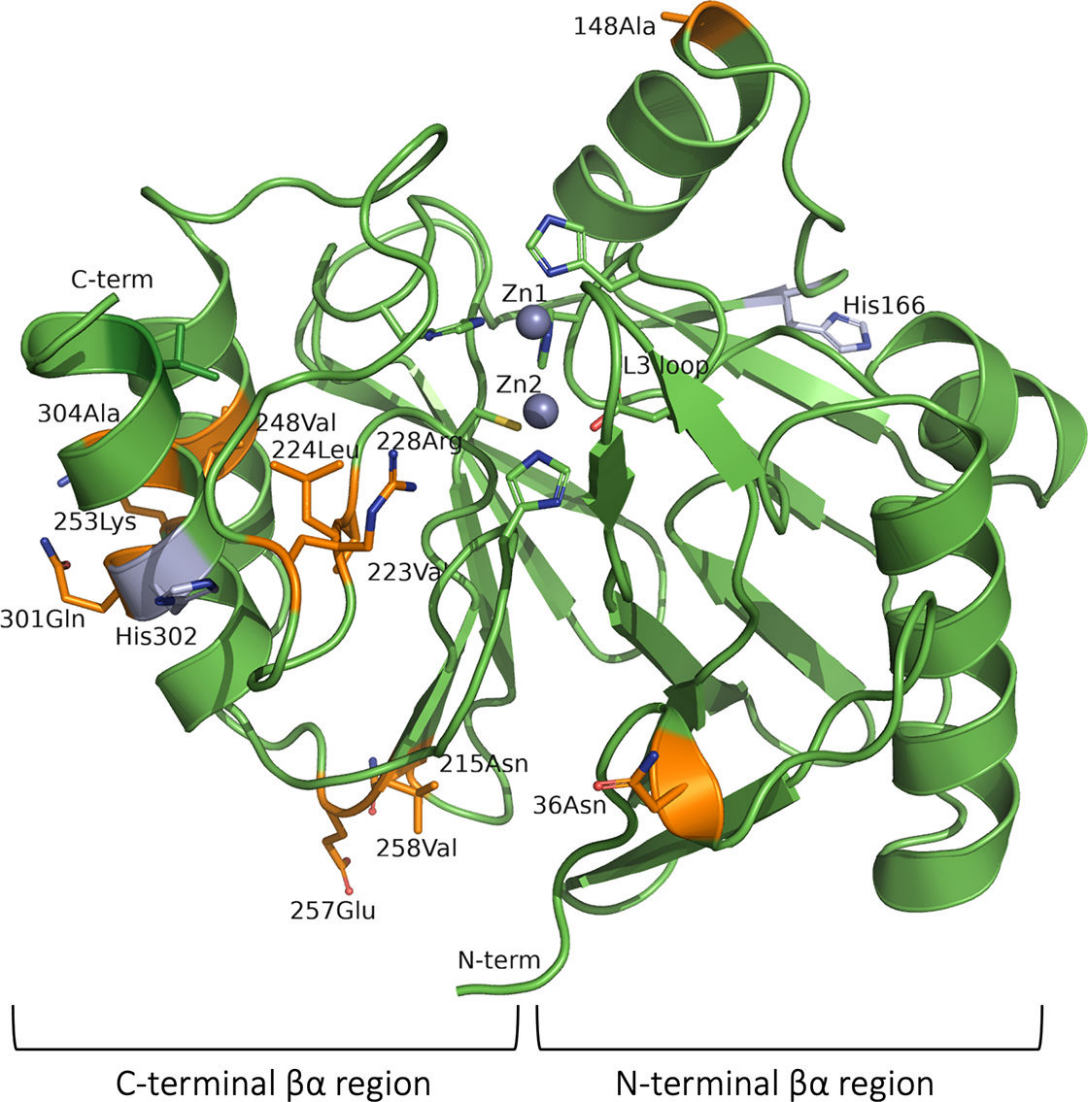
Substituted residues between VIM-1 and VIM-2 are shown in orange in the VIM-28 structure.

There are 25 amino acid substitutions between VIM-1 and VIM-2, most of which occur in the C-terminal βα region of αββα-fold (Fig. 4). The residues at the N- and C-terminal ends are also substituted (residues 24, 26-28, 311, and 316). Substituted residues are often exposed to the solvent (positions 36, 148, 253, 301, and both N- and C-terminal ends), simultaneously at several residues that are structurally close to one another. We constructed VIM-28 variants with the residues substituted with those of VIM-2 to analyze their kinetic properties. These amino acid substitutions had only minor effects (Table S2).

## DISCUSSION

The active site of B1 MBLs is a shallow groove flanked by two loops, L3 and L10, which span from residues 59–65 and 223–241, respectively. These loops contain residues that interact with substrates (11, 19). In many B1 enzymes, the L3 loop contains a hydrophobic (often aromatic) residue in position 64 that establishes hydrophobic contact with substrates. In addition, hydrophobic residues in positions 61 and 67, located at the base of the loop, form a hydrophobic patch that interacts with R1 substituents of β-lactams. The L10 loop often contains a Lys residue at position 224, which is critical for binding the C3/C4 carboxyl group of substrates, together with the interaction with the Zn2 site. In VIM-2, an Arg residue at position 228 has been proposed to substitute for Lys224 in substrate binding. VIM enzymes exhibit variability in the residues at positions 224 and 228 in the L10 loop (Table 1). In this study, we analyzed the kinetic properties and thermal stability of VIM-28 and its close variants. Subsequently, we assessed the susceptibility profiles of the cells expressing these enzymes and analyzed the crystal structure of VIM-28.

Kinetic analysis of the VIM-4 revealed that the L224H substitution alone did not drastically change the kinetic parameters, except for a slight increase in catalytic efficiency for cephalosporins and meropenem. In contrast, the R228S substitution increased *K*_m_ values toward the tested substrates in VIM-26 (R228S) and VIM-1 (L224H/R228S). This result is consistent with the structural studies of enzyme–inhibitor complexes in IMP-1 and VIM-2, demonstrating the importance of Arg228 for the binding of the C3/C4 carboxyl group in the substrate as a substitute for Lys224 of IMP-1 (18, 20). Leiros et al. found that VIM-26 exhibits higher substrate affinity toward penicillins than VIM-1 (21); however, our results do not align with these findings, possibly because of methodical differences in the kinetic analysis and/or preparation of enzymes. In our study, the increase in the *K*_m_ values observed for VIM-26 was partly mitigated by the L224H substitution in VIM-1. These data suggested that the presence of a positively charged residue at either position 224 or 228 enhanced electrostatic interaction with the C3/C4 carboxylate group of the substrates. Notably, the R228S substitution increased *K*_m_ toward meropenem by one order of magnitude in both VIM-26 and VIM-1, reducing catalytic efficiency. These data demonstrate that the Arg residue at position 228 plays an important role in meropenem binding, perhaps not only in the electrostatic interaction with its C3 carboxylate group but also through different specific interactions with its R2 group with a pyrroline ring. The catalytic efficiency of VIM-1 (L224H/R228S) for ceftazidime was five times higher than that of VIM-28 and four times higher than VIM-4 (L224H). However, no increase in *k*_cat_/*K*_m_ was observed in VIM-26 (R228S), indicating that the combination of the R228S and L224H substitutions was required for increased efficiency in ceftazidime hydrolysis. In the kinetic study of VIM-13, the same combination of substitutions resulted in properties similar to those of VIM-1, although the catalytic efficiency toward ceftazidime was less than that observed for VIM-28 (22). Steric hindrance and/or electrostatic repulsion may have occurred between the R2 group with a positively charged pyridine ring and the Arg228 side chain of VIM-28 when ceftazidime bound to VIM-28. The study of VIM-24, an R228L variant derived from VIM-2, demonstrated that the R228L substitution increases catalytic efficiency toward ceftazidime and cefepime, while decreasing catalytic efficiency toward other substrates tested (23). These findings further support the functional importance of residue 228 in determining the substrate specificity profile not only in VIM-1-type enzymes but also in VIM-2-derived enzymes.

Overall, the susceptibility profiles of the cells producing the enzymes were consistent with the results of the kinetic analysis. The L224H/R228S variant exhibited an eightfold increase in ceftazidime MIC compared with VIM-28, likely reflecting the fivefold increase in catalytic efficiency toward ceftazidime.

Zinc ions are essential not only for the activity but also for the stability of MBLs. Some MBLs have evolved to maintain their activity and/or stability under Zn(II) deprivation, often associated with their zinc binding affinity (11). In this context, DPA, a metal-chelating agent that can mimic the effect elicited by the action of calprotectin, has been used to evaluate the resistance phenotype of MBL-producing bacteria under zinc-limiting conditions (15, 24). Our relative MIC results revealed that cells producing VIM-26 (R228S) were similarly or more resistant to DPA and EDTA in comparison with those producing VIM-28, indicating that this substitution may enhance the zinc binding affinity of the enzyme. In contrast, cells producing VIM-4 (L224H), VIM-1 (L224H/R228S), and the VIM-28 V223H/L224Y variant displayed decreased resistance, suggesting that substitutions at position 224 impair the metal-binding affinity of VIM-28. These findings indicate that the introduction of positively charged residues at positions 224 and 228 may weaken zinc coordination, possibly by disrupting the hydrogen-bonding network surrounding the zinc ions or by inducing electrostatic repulsion.

To further investigate the effects of these substitutions on enzyme stability under different zinc conditions, DSF assays were performed in the presence of 0–50 μM ZnCl_2_. The Zn content of the enzyme preparations used for these measurements was not determined in this study. The *T*_m_ was highest in the absence of zinc ions and decreased with increasing zinc concentration in all the tested enzymes. A lower *T*_m_ in the presence of zinc ions was also reported in VIM-7, suggesting the presence of one or more nonspecific zinc-binding sites outside the active-site region that destabilizes the protein (25). It might also explain the lower *T*_m_ in the presence of zinc ions. VIM-26 (R228S) exhibited the highest *T*_m_ among all the tested enzymes, higher than that of VIM-28 by 3.5°C. In contrast, the enzymes with a histidine residue at position 224 showed a lower *T*_m_ than VIM-28, with VIM-4 exhibiting the lowest *T*_m_, lower than that of VIM-28 by 7.5°C. The *T*_m_ of the V223I/L224Y variant was also lower than that of VIM-28 by 2.5°C. These results indicate that the R228S substitution increases the thermal stability of the enzyme, whereas the substitutions at Leu224 reduce it. These results, in agreement with the relative MIC data, suggest that positively charged residues at positions 224 and 228 may reduce the thermal stability, possibly by affecting zinc binding. Increased thermal stability has been reported for some VIM and NDM variants (25–27), which could increase the intracellular longevity of the enzyme and improve antibiotic resistance in enzyme-producing bacteria.

The result of the structural analysis signifies the possible dimer formation of VIM-28 through the L3 loop (Fig. 2A). This intermolecular interaction observed in VIM-28 may be a crystallographic artifact because no dimers were detected on size-exclusion chromatography (data not shown). However, such pseudo-dimer formation may easily occur in some MBLs since the formation of the four-stranded antiparallel β-sheet through the same β-hairpin structure was often observed in VIM-type enzymes and also in other B1 MBLs, such as IMP-18 (28).

The crystal structure of VIM-28 contains one citrate anion and two zinc ions (Zn1 and Zn2) bound to the active site (Fig. 3A). The electron density of Zn2 is weaker than that of Zn1, indicating a lower zinc affinity for the Zn2 binding site than for the Zn1 site, as reported previously in the VIM-1 study (17). A low affinity for this site was also implied by the fluctuating position of the Zn2 ion among the VIM enzyme structures (Fig. 3B). The structural superposition of the VIM enzymes showed that Zn1 overlaps well with a difference in the position within 0.3 Å. However, a higher variation was observed in the position of Z2, and a large deviation of 1.2 Å was observed between VIM-17 (5MM9) and VIM-26 (4UWO). Cys221 oxidation was not observed in VIM-28, whereas the Cys221 side chain was clearly oxidized in the absence of Zn2 in the crystal structures of some VIM enzymes, such as VIM-2, VIM-26, and VIM-31 (13, 21, 29). Although the factors deciding the oxidation/reduction state of Cys221 are unclear and remain to be investigated, it might reflect the difference in the zinc-binding affinity of the Zn2 site.

There are 25 amino acid substitutions between VIM-1 and VIM-2. We constructed VIM-28 variants by replacing these residues with those of VIM-2 and analyzed their kinetic properties; however, the amino acid substitutions did not cause drastic changes in their kinetic properties (Table 2 and Table S2). Among these variants, the A304T substitution decreased the catalytic efficiency of the enzyme toward penicillins and cephalothin. Although this residue is located away from the active center, the substitution may have disrupted the small hydrophobic core formed between two α-helices in the C-terminal domain (Fig. 4). One of these α-helices has His302 and Thr303 whose side chains are hydrogen-bonded to the main chain atoms of the L10 loop. These hydrogen bonds may stabilize the conformation of the L10 loop, as seen in other VIM enzymes, such as VIM-1 (Fig. 3B). The A304T substitution may negatively impact the positioning of the residues in the L10 loop. In the V223I/L224Y/V248I variant, lower *k*_cat_ and *k*_cat_/*K*_m_ values were observed for penicillin G, cefotaxime, and meropenem, whereas the *k*_cat_/*K*_m_ toward ceftazidime increased threefold. This change may have resulted from the V223I/V224Y substitution conferring similar kinetic properties. The side chains of these residues are close to each other in the three-dimensional structure, and mutations in these positions may affect the immediate environment of the substrate binding site and the orientation and/or flexibility of Cys221 in the same L10 loop. Although VIM-2 can hydrolyze carbapenems more efficiently than VIM-1 (12), the amino acid substitutions in these positions have little effect on the kinetic properties of VIM-28.

The VIM enzymes constitute one of the largest groups in B1 MBLs. Our kinetic analyzes indicated that substitutions at position 224 resulted in catalytic activities similar to those of VIM-28 toward all the tested substrates; however, the catalytic activity of VIM-28 was higher toward many substrates than the enzymes carrying the R228S substitution, except for the higher activity of VIM-1 (L224H/R228S) toward ceftazidime. Our analyses revealed zinc-dependent effects on both enzyme stability and resistance phenotypes in enzyme-producing cells. While *T*_m_ was highest in the absence of zinc ions and decreased with increasing zinc concentration in all tested enzymes, relative MIC measurements demonstrated that the R228S substitution enhanced zinc-binding affinity of the enzyme, whereas substitutions at position 224 impaired it. Overall, VIM-28 not only exhibits high catalytic activity toward a wide range of antibiotics except for ceftazidime but also has higher zinc-binding affinity and higher thermal stability than its putative ancestor VIM-4. These properties likely confer enhanced resistance to zinc deprivation on the cells producing VIM-28, possibly contributing to prolonged functionality under zinc-limited conditions. Although further investigation is required to understand the properties of VIM-28 in detail, including the susceptibility to inhibitors, the increased stability and zinc affinity observed in this enzyme may represent an evolutionary driving force in MBLs, including VIM-type enzymes (26, 27).

## MATERIALS AND METHODS

### Construction of the VIM-28 expression vector

The gene encoding VIM-28 was amplified by PCR, using genomic DNA extracted from *P. aeruginosa* as template and the following primers: VIM28-F (5′-CCGGCATATGTTAAAAGTTATTAGTAGTTTATTGGTCTAC-3′), with an *Nde*I restriction site (underlined) at the position of the starting Met codon, and VIM28-R (5′-GGATCCCTACTCGGCGACTGAG-3′), with a *Bam*HI restriction site (underlined) after the stop codon. The amplified fragment was inserted into a pCR2.1-TOPO vector using a TA cloning kit (Invitrogen, Carlsbad, CA, USA). Subsequently, *Escherichia coli* TOP10 (Invitrogen) was transformed using this plasmid. For the overexpression of VIM-28, the region encoding the mature VIM-28 was amplified with the primers vim28-remove-ss (5′-GCGTCTGTCATGGCTCATATGAGTCCGTTAGCCCAT-3′), which had an *Nde*I restriction site (underlined) at the 5′-end of the gene, and T7 terminator (5′-ATGCTAGTTATTGCTCAGCGG-3′). The amplicon was digested with *Nde*I and *Bam*HI and inserted into expression vector pET-9a to construct pET9a-vim28Δss.

### Overproduction and purification of VIM-28

*E. coli* BL21(DE3) pLysS (Invitrogen) harboring pET9a-vim28Δss was cultured in 100 mL 2×TY medium with 20 µg/mL kanamycin at 37°C. When the absorbance at 600 nm reached approximately 0.5, isopropyl β-d-1-thiogalactopyranoside was added to a final concentration of 0.1 mM for the induction of gene expression. After further overnight cultivation at 22°C, the cells were harvested by centrifugation, resuspended in 20 mM HEPES buffer (pH 7.5) containing 50 µM ZnCl_2_, and disrupted by sonication. The supernatant was loaded on a 1-mL HiTrap DEAE FF column (Cytiva, Marlborough, MA, USA) equilibrated with 20 mM HEPES buffer (pH 7.5) containing 50 µM ZnCl_2_. After protein elution with a NaCl linear gradient concentration of 0–500 mM, the target protein was concentrated with Amicon Ultra-4 (Merck Millipore, Burlington, MA, USA) and applied to a Superdex75 100/300 GL size-exclusion column (Cytiva) equilibrated with 20 mM HEPES buffer (pH 7.5) containing 150 mM NaCl and 50 µM ZnCl_2_. The purified protein was again concentrated using Amicon Ultra-4, and its purity was verified with SDS-PAGE with 15% acrylamide gel to be approximately 95% or higher (Fig. S2).

### Construction of expression vectors to produce VIM enzymes by site-directed mutagenesis

The expression vectors for the VIM enzymes were constructed by site-directed mutagenesis with the PrimeSTAR Mutagenesis Basal Kit (Takara Bio Co., Shiga, Japan) using the pET9a-vim28Δss plasmid as template. The oligonucleotide primers used for the introduction of the mutations are listed in Table S3. The VIM enzymes were produced and purified as described above.

### Kinetic assays

The antibiotics and chemicals used for steady-state kinetic analysis are as follows: ampicillin (Δε_235_ = −820 M^−1^cm^−1^) and cefotaxime (Δε_260_ = −7,500 M^−1^cm^−1^) were purchased from FUJIFILM Wako Chemicals (Tokyo, Japan). Benzylpenicillin (Δε_235_ = −775 M^−1^cm^−1^), cephaloridine (Δε_260_= −10,000 M^−1^cm^−1^), ceftazidime (Δε_260_ = −9,000 M^−1^cm^−1^), imipenem (Δε_300_ = −9,000 M^−1^cm^−1^), and meropenem (Δε_300_ = −6,500 M^−1^cm^−1^) were purchased from Sigma-Aldrich (St. Louis, MO, USA). Kinetic analysis was performed as described by Borgianni et al. (30). An enzymatic reaction was performed at 30°C in 500 µL of 20 mM HEPES buffer (pH 7.5) containing 50 µM ZnCl_2_ using a V-530 spectrophotometer (JASCO Co., Tokyo, Japan). The enzyme used for the assays was diluted with HEPES buffer containing 20 µg/mL bovine serum albumin. At least three independent progress curves were obtained for each substrate until reproducible results were obtained.

### Susceptibility profiles

The genes encoding VIM-28 and its related enzymes were inserted into pMW119_Kan^R^_Ptac, a low-copy cloning vector constructed from pMW119 (31). Plasmid-bearing *E. coli* DH5α cells were cultured in cation-adjusted Mueller–Hinton broth (Becton, Dickinson and Company, Franklin Lakes, NJ, USA). Antibiotic susceptibility testing was performed by microbroth dilution according to the Clinical and Laboratory Standards Institute guidelines (32, 33). Quality control was performed with *E. coli* ATCC 25922 (NBRC 00015034) and *P. aeruginosa* ATCC 27853, which was kindly provided by Dr. Go Yamamoto, with MICs falling within the expected concentration ranges.

Relative MICs were calculated as (MIC_enzyme_ – MIC_control_)/(MIC_enzyme + 0 μM DPA/EDTA_ – MIC_control + 0 μM DPA/EDTA_) (15). Here, MIC_enzyme_ refers to values measured for *E. coli* DH5α carrying the pMW119_Kan^R^_Ptac plasmid with an inserted gene encoding VIM-28 or its related enzymes, and MIC_control_ refers to values measured for *E. coli* DH5α carrying the plasmid without the insertion, under each set of conditions. MIC_enzyme + 0 μM DPA/EDTA_ and MIC_control + 0 μM DPA/EDTA_ indicate the corresponding values obtained in media without the addition of metal-chelating agent.

### DSF assay

The purified enzyme was applied to a PD-10 column (Cytiva) equilibrated with 20 mM Tris-H_2_SO_4_ buffer (pH 7.5), and the collected fraction was concentrated using the 10 K Amicon Ultra-4 to obtain a final concentration of 5.0 mg/mL. DSF was performed using a CFX96 Touch Real-Time PCR Detection System (BIO-RAD, Hercules, CA, USA). Each well of a 96-well PCR plate was filled with a 25-µL solution containing 5× SYPRO-Orange (Invitrogen), 20 mM Tris-H_2_SO_4_ (pH 7.5), 0–50 µM ZnCl_2_, and 1.0 mg/mL purified enzyme. The analysis was performed in FRET scan mode (excitation: 470 mm; emission: 570 nm) with increasing temperature from 25°C to 95°C at a rate of 0.5°C/s. At least three independent measurements were performed for each condition. Data were analyzed using GraphPad Prism ver. 5.01 (GraphPad Software, Boston, MA, USA).

### Crystallization, X-ray data collection, and structural determination

For crystallization, purified VIM-28 was concentrated to 10 mg/mL with the Amicon Ultra-4 (10 kDa MWCO) using 5 mM HEPES (pH 7.5). Initial screening of crystallization conditions was performed by the hanging-drop vapor-diffusion method at 283 and 289 K using Crystal Screen and Crystal Screen II from Hampton Research (Aliso Viejo, CA, USA). The crystallization conditions were further optimized, and crystals suitable for data collection were obtained with the reservoir solution containing 1.4 M ammonium citrate (pH 6.5) at 289 K. X-ray diffraction data were collected at beamline BL5A of Photon Factory, KEK (Tsukuba, Japan). The diffraction patterns were indexed, integrated, and scaled using iMosflm and the CCP4 suite programs (34, 35). The structure of VIM-4 (PDB entry code: 2WHG) was used as a model for molecular replacement with MOLREP (36). The model was further refined using COOT and Refmac5 (37, 38).

## Data Availability

The authors confirm that the data supporting the findings of this study are available within the article and its supplementary materials. The structure as well as the corresponding structure factor amplitudes of VIM-28 is available at the RCSB PDB under accession code 7YRP.

## SUPPLEMENTAL MATERIAL

Supplemental material spectrum.02788-25-s0001.docx Tables S1 to S3; Fig. S1 and S2.

ASM does not own the copyrights to Supplemental Material that may be linked to, or accessed through, an article. The authors have granted ASM a non-exclusive, world-wide license to publish the Supplemental Material files. Please contact the corresponding author directly for reuse.
