# Supplementary material for: Biochemical and structural analyses of metallo-β-lactamase VIM-28: impact of substitutions at residues 224 and 228 on substrate profile, stability, and zinc affinity
Source: Microbiol Spectr. 2026 Feb 9;14(3):e02788-25. doi: 10.1128/spectrum.02788-25 (PMC12955476; doi:10.1128/spectrum.02788-25)
Supplement: Supplemental material — Tables S1 to S3; Fig. S1 and S2. [file spectrum.02788-25-s0001.docx]

**Supplementary data:**

**Biochemical and structural analyses of Metallo-β-lactamase VIM-28: impact of substitutions at residues 224 and 228 on substrate profile, stability, and zinc affinity**

*Hiromu Sato^1^, Nao Ishizawa^1^, Ryo-ya Koto^1^, Kaisei Hiura^1^, Hiyori Saito^1^, Yoshiki Kato^1,2^, Nancy D. Hanson^3^, Yoshikazu Ishii^4^, and Akiko Shimizu-Ibuka^1, 2,#^*

^1^ Department of Applied Life Sciences, Niigata University of Pharmacy and Applied Life Sciences, 265-1 Higashijima, Akiha-ku, Niigata 956-8603, Japan

^2^ Graduate School of Science, Kanagawa University, 3-27-1 Rokkakubashi, Kanagawa-ku, Yokohama, Kanagawa 221-8686, Japan

^3^ Department of Medical Microbiology and Immunology, Creighton University, 2500 California Plaza, Omaha, Nebraska 68178, USA

^4^ Department of Microbial Genomics and Ecology, Center for the Planetary Health and Innovation Science, The IDEC Institute, Hiroshima University, 1-3-2 Kagamiyama, Higashi-Hiroshima City, Hiroshima 739-8511, Japan

^#^Address correspondence to Akiko Shimizu-Ibuka, ibuka@kanagawa-u.ac.jp

E-mail: ibuka@kanagawa-u.ac.jp

**Contents:**

**Table S1.** Data collection and structural refinement

**Table S2.** Kinetic parameters of VIM-28 and its variants.

**Table S3.** Primer sequences used for the introduction of the mutations.

**Fig. S1.** Thermal stability measurements.

**Fig. S2.** SDS-PAGE analysis of VIM-28 purification.

**Table S1.** Data collection and structural refinement

| Data collection Statistics |  |
| --- | --- |
| Unit cell dimensions (Å) | *a* = 139.36°, *b* = 45.91°, *c* = 103.26°  α = γ = 90°, β = 103.59° |
| Space group | *C121* |
| Resolution (Å) | 67.73–2.00 (2.11–2.00) |
| Number of observed reflections | 135387 (19672) |
| Number of unique reflections | 43247 (6218) |
| Completeness | 99.7 (99.7) |
| Multiplicity | 3.1 (3.2) |
| *I*/σ(*I*) | 8.3 (2.4) |
| R_sym_ | 0.102 (0.494) |
| Refinement Statistics |  |
| Resolution (Å) | 63.48–2.00 (2.05–2.00) |
| Number of reflections | 41098 (2991) |
| Number of atoms |  |
| Protein | 3395 |
| Water | 244 |
| Citrate ion | 26 |
| Zinc ion | 4 |
| Glycerol | 36 |
| R-factor (R-work) | 0.173 (0.257) |
| R-factor (R_free_) | 0.204 (0.276) |
| Average B factors (Å^2^) |  |
| Protein | 32.0 |
| Water | 35.5 |
| Ligands | 39.4 |
| RMSD from ideal |  |
| Bond lengths (Å) | 0.007 |
| Bond angles (deg) | 1.39 |
| Ramachandran plot (%) |  |
| Favored | 97.8 |
| Allowed | 2.0 |
| Outliers | 0.2 |

RMSD, root mean square deviation.

|  | Ampicillin | Penicillin G | Cephalothin | Cefotaxime | Ceftazidime | Meropenem |
| --- | --- | --- | --- | --- | --- | --- |
| VIM-28 |  |  |  |  |  |  |
| *k*_cat_ (s^−1^) | 191 ± 36 | 1240 ± 80 | 276 ± 13 | 285 ± 9 | NA^a^ | 20.6 ± 1.2 |
| *K*_m_ (μM) | 12.5 ± 4.9 | 176 ± 12 | 9.66 ± 0.79 | 27.2 ± 1.8 | >400 | 14.3 ± 0.9 |
| *k*_cat_/*K*_m_ (s^−1^μM^−1^) | 15.3 | 7.03 | 28.6 | 10.5 | 0.0359^b^ ± 0.0034 | 1.44 |
| H24F/G26V/E27D/P28S (N-term) |  |  |  |  |  |  |
| *k*_cat_ (s^−1^) | 212 ± 34 | 1290 ± 50 | 296 ± 12 | 322 ± 17 | NA^a^ | 18.6 ± 0.5 |
| *K*_m_ (μM) | 19.2 ± 3.3 | 159 ± 13 | 11.0 ± 0.8 | 28.4 ± 1.5 | >400 | 13.1 ± 2.0 |
| *k*_cat_/*K*_m_ (s^−1^μM^−1^) | 11.0 | 8.11 | 27.0 | 11.3 | 0.0337^b^ ± 0.0031 | 1.42 |
| A148V |  |  |  |  |  |  |
| *k*_cat_ (s^−1^) | 186 ± 25 | 1230 ± 20 | 292 ± 15 | 355 ± 18 | NA^a^ | 22.8 ± 0.4 |
| *K*_m_ (μM) | 15.8 ± 3.8 | 171 ± 9 | 11.1 ± 1.0 | 33.1 ± 1.3 | >400 | 13.6 ± 0.9 |
| *k*_cat_/*K*_m_ (s^−1^μM^−1^) | 11.8 | 7.21 | 26.2 | 10.7 | 0.0328^b^ ± 0.0032 | 1.68 |
| V248I |  |  |  |  |  |  |
| *k*_cat_ (s^−1^) | 206 ± 29 | 1410 ± 40 | 297 ± 28 | 383 ± 28 | NA^a^ | 21.1 ± 1.4 |
| *K*_m_ (μM) | 16.2 ± 4.1 | 179 ± 11 | 9.80 ± 0.74 | 31.4 ± 1.5 | >400 | 13.6 ± 0.5 |
| *k*_cat_/*K*_m_ (s^−1^μM^−1^) | 12.7 | 7.89 | 30.3 | 12.2 | 0.0363^b^ ± 0.0030 | 1.56 |
| A304T |  |  |  |  |  |  |
| *k*_cat_ (s^−1^) | 163 ± 29 | 1110 ± 20 | 240 ± 14 | 284 ± 21 | NA^a^ | 19.5 ± 1.5 |
| *K*_m_ (μM) | 16.4 ± 4.2 | 197 ± 19 | 10.3 ± 0.7 | 27.6 ± 1.9 | >400 | 12.7 ± 1.3 |
| *k*_cat_/*K*_m_ (s^−1^μM^−1^) | 9.91 | 5.60 | 23.2 | 10.3 | 0.0276^b^ ± 0.0024 | 1.54 |
| K251Q |  |  |  |  |  |  |
| *k*_cat_ (s^−1^) | 191 ± 18 | 1370 ± 60 | 289 ± 6 | 352 ± 11 | NA^a^ | 22.5 ± 0.9 |
| *K*_m_ (μM) | 16.9 ± 4.9 | 199 ± 12 | 11.0 ± 0.6 | 34.6 ± 1.3 | >400 | 14.4 ± 1.1 |
| *k*_cat_/*K*_m_ (s^−1^μM^−1^) | 11.3 | 6.87 | 26.3 | 10.1 | 0.0313^b^ ± 0.0025 | 1.56 |
| V258F |  |  |  |  |  |  |
| *k*_cat_ (s^−1^) | 178 ± 16 | 1280 ± 44 | 260 ± 13 | 305 ± 14 | NA^a^ | 23.0 ± 1.9 |
| *K*_m_ (μM) | 14.2 ± 2.4 | 186 ± 9 | 10.1 ± 0.6 | 30.5 ± 1.8 | >400 | 14.0 ± 0.7 |
| *k*_cat_/*K*_m_ (s^−1^μM^−1^) | 12.5 | 6.93 | 25.8 | 10.0 | 0.0333^b^ ± 0.0039 | 1.64 |
| E257Q/V258F |  |  |  |  |  |  |
| *k*_cat_ (s^−1^) | 177 ± 39 | 1200 ± 100 | 274 ± 8.9 | 302 ± 24 | NA^a^ | 20.1 ± 1.4 |
| *K*_m_ (μM) | 17.1 ± 3.9 | 159 ± 10 | 10.3 ± 0.4 | 32.2 ± 3.0 | >400 | 11.9 ± 1.0 |
| *k*_cat_/*K*_m_ (s^−1^μM^−1^) | 10.4 | 7.56 | 26.7 | 9.41 | 0.0331^b^ ± 0.0042 | 1.69 |
| N215S/E257Q/V258F |  |  |  |  |  |  |
| *k*_cat_ (s^−1^) | 173 ± 36 | 1100 ± 60 | 219 ± 13 | 284 ± 18 | NA^a^ | 18.7 ± 1.1 |
| *K*_m_ (μM) | 14.0 ± 5.0 | 137 ± 14 | 10.0 ± 0.4 | 31.2 ± 1.2 | >400 | 12.9 ± 0.9 |
| *k*_cat_/*K*_m_ (s^−1^μM^−1^) | 12.4 | 8.02 | 21.9 | 9.11 | 0.0330^b^ ± 0.0037 | 1.45 |
| V248I/Q301K/A304T |  |  |  |  |  |  |
| *k*_cat_ (s^−1^) | 143 ± 19 | 1070 ± 10 | 245 ± 15 | 280 ± 5.4 | NA^a^ | 16.5 ± 0.5 |
| *K*_m_ (μM) | 15.7 ± 5.5 | 166 ± 4 | 10.3 ± 0.3 | 33.9 ± 1.3 | >400 | 13.1 ± 1.0 |
| *k*_cat_/*K*_m_ (s^−1^μM^−1^) | 9.14 | 6.44 | 23.8 | 8.24 | 0.0260^b^ ± 0.0015 | 1.26 |
| K311T/A316V (C-term) |  |  |  |  |  |  |
| *k*_cat_ (s^−1^) | 245 ± 75 | 1350 ± 50 | 363 ± 19 | 400 ± 9 | NA^a^ | 22.5 ± 1.1 |
| *K*_m_ (μM) | 18.0 ± 4.3 | 174 ± 22 | 11.2 ± 0.4 | 38.0 ± 1.7 | >400 | 14.1 ± 1.0 |
| *k*_cat_/*K*_m_ (s^−1^μM^−1^) | 13.2 | 7.73 | 32.3 | 10.5 | 0.0310^b^ ± 0.0031 | 1.60 |

**Table S2.** Kinetic parameters of VIM-28 and its variants

^a^ Not available; *K*_m_ too high to determine accurately.

^b^ The *k*_cat_/*K*_m_ ratio was calculated from the initial slope using *v* = *k*_cat_/*K*_m_([S]).

**Table S3.** Primer sequences used for the introduction of the mutations.

| Mutation | Primer | Sequence (5′→3′) |
| --- | --- | --- |
| H24F/G26V/E27D/P28S (N-term) | VIM28-Nterm-for | TTCTCCGTCGACTCGAGTGGTGAGTATCCGACA |
|  | VIM28-Nterm-rev | CGAGTCGACGGAGAAGGCTAACGGACTCATATG |
|  | VIM28ss-Nterm-rev | CGAGTCGACGGAGAAGGCTAACGGACTTGCGAC |
| A148V | VIM-28 A148V For | GCTGGCCGAGGTAGAGGGGAACGAGATTCC |
|  | VIM-28 A148V Rev | TCTACCTCGGCCAGCCGGCGTGTCGACGGT |
| N215S | VIM-28-N215S-for | AGCGTCAGTACTATACGGTGGTTGTGCC |
|  | VIM-28-N215S-rev | TATAGTACTGACGCTGACGGGACGTATAC |
| L224H (VIM-4) | VIM-28-L224H FOR | GTTCATGAGCTCTCACGCACGTCTGCGGGG |
|  | VIM-28-L224H REV | TGAGAGCTCATGAACGGCACAACCACCGT |
| R228S | VIM28-R228S-for | TGTCAAGTACTTCTGCGGGGAACGTGGCC |
|  | VIM28-R228S-rev | CAGAAGTACTTGACAACTCAAGAACGGC |
| V223I+L224Y | VIM28-V223I+L224Y-for | CATTTATGAGCTCTCACGCACGTCTGCGGGG |
|  | VIM28-V223I+L224Y-rev | GAGAGCTCATAAATGGCACAACCACCGTATAG |
| V248I | VIM28-V248I-for | CACCTCGATCGAGCGGATTCAAAAACAC |
|  | VIM28-V248I-rev | CGCTCGATCGAGGTGGGCCATTCAGCC |
| K251Q | VIM-28 K253Q For | CGGATCCAACAACACTACCCGGAAGCAG |
|  | VIM-28 K253Q Rev | GTGTTGTTGGATCCGCTCAACGGAGGTG |
| V258F | VIM28-V258F-for | AAGCAGAATTCGTCATTCCCGGGCACG |
|  | VIM28-V258F-rev | TGACGAATTCTGCTTCCGGGTAGTGT |
| E257Q/V258F | VIM28-E257Q/V258F-for | GGAAGCACAATTCGTCATTCCCGGGC |
|  | VIM28-E257Q/V258F-rev | ACGAATTGTGCTTCCGGGTAGTGTTT |
| A304T | VIM28-A304T-for | CTGCAGCACACAACGAACGTTGTTAAAGCAC |
|  | VIM28-A304T-rev | CGTTGTGTGCTGCAGCAAGTCTAGACCGCCC |
| Q301K/A304T | VIM28-Q301K/A304T-for | CTTAAGCACACTACGAACGTTGTCAAAGCAC |
|  | VIM28-Q301K/A304T-rev | CGTAGTGTGCTTAAGCAAGTCTAGACCGCCC |
| K311T/A316V (C-term) | VIM28-K311T/A316V-for | CAAATCGATCGGTCGTCGAGTAGGGATCCGGCTG |
|  | VIM28-K311T/A316V-rev | ACGACCGATCGATTTGTGTGTGCTTTGACAACGT |


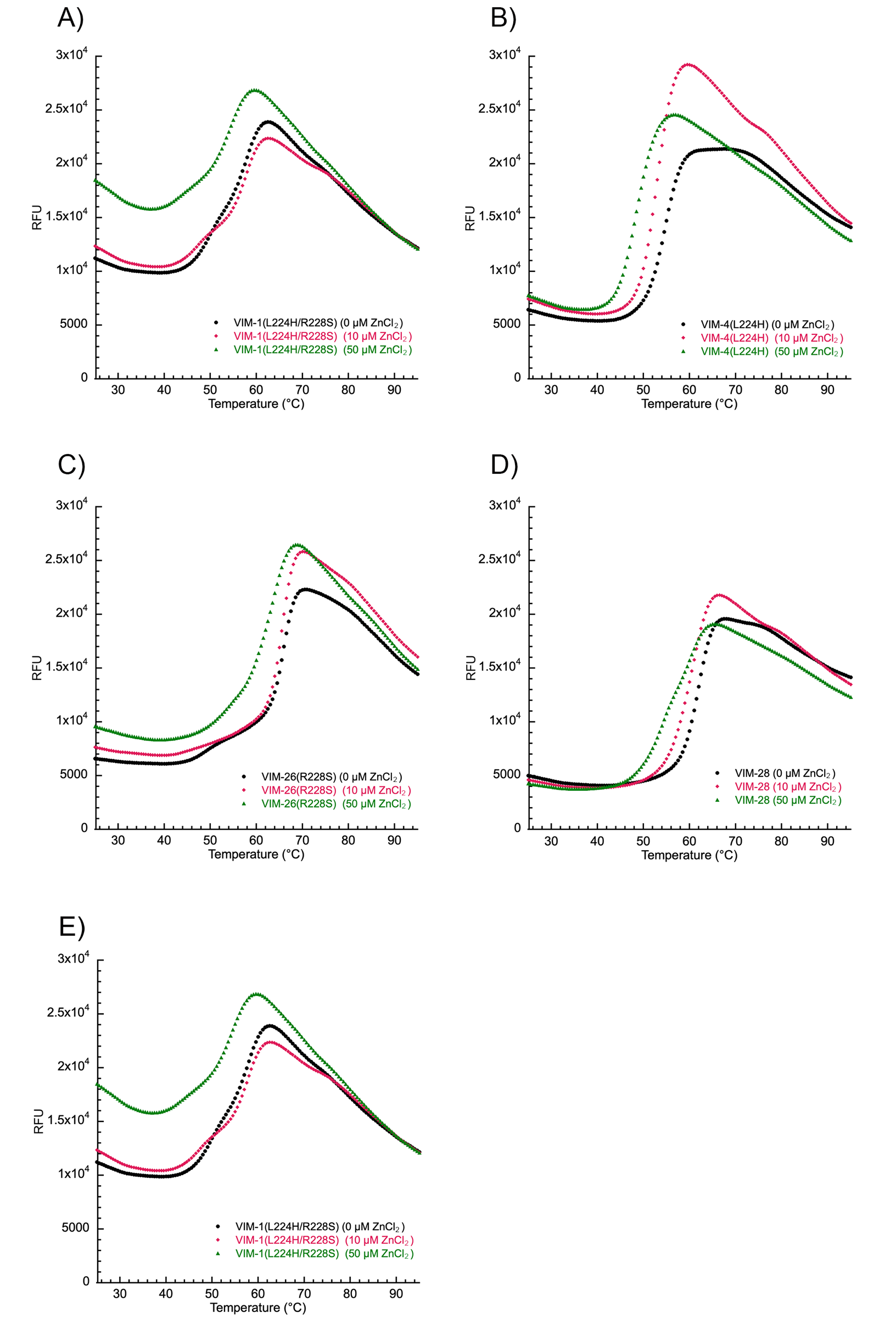


**Fig. S1.** Thermal stability measurements. The relative fluorescence units (RFU) of SYPRO Orange measured as a function of temperature. Data shown in black are measured in the absence of zinc ions, whereas data in magenta and in green are measured in the presence of 10 μM and 50 μM ZnCl_2_, respectively.


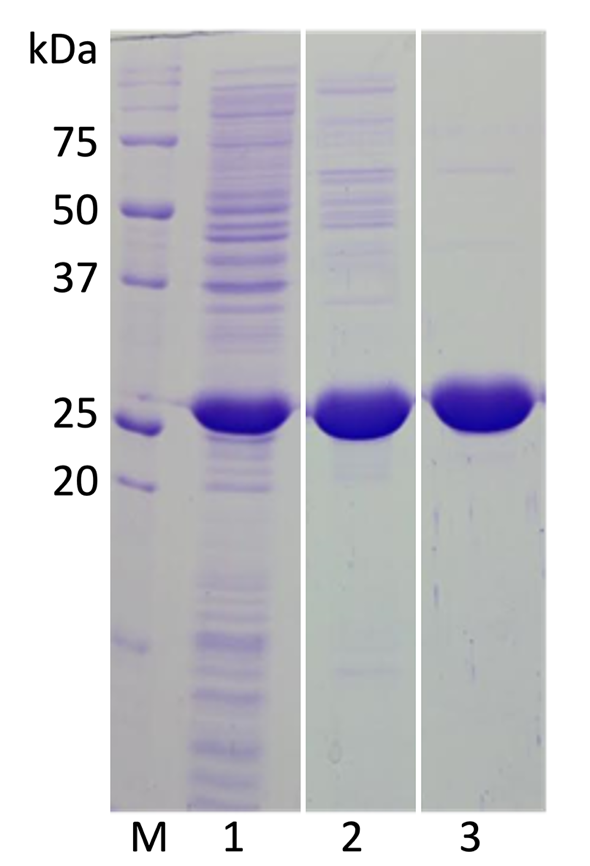


**Fig. S2.** SDS-PAGE analysis of VIM-28 purification. Lanes: M, molecular weight marker; 1, crude extract; 2, after DEAE ion-exchange chromatography; 3, after size-exclusion chromatography.
